# Differences in the Biliary Microbiome Between Biliary Tract Cancer and Benign Biliary Disease

**DOI:** 10.3390/microorganisms14010208

**Published:** 2026-01-16

**Authors:** Hye Ji Lee, Sung Hee Park, Sung Yong Han, Jong Hyun Lee, Dong Uk Kim, Hyung Il Seo

**Affiliations:** 1 Biomedical Research Institute, Pusan National University Hospital, Pusan 49241, Republic of Korea; lhj4151199@naver.com (H.J.L.); scaletlee@hanmail.net (S.H.P.); keiasikr@nate.com (J.H.L.); seohi71@hanmail.net (H.I.S.); 2 Department of Internal Medicine, Pusan National University College of Medicine, Yangsan 50612, Republic of Korea; 3 Department of Internal Medicine, Gumi Medical Center, CHA University, Gumi 39233, Republic of Korea; 4 Department of Surgery, Pusan National University College of Medicine, Yangsan 50612, Republic of Korea

**Keywords:** biliary tract cancer, biliary benign disease, bile, bacteroides

## Abstract

Bile contains many bacteria that can contribute to various diseases. Therefore, identifying bile microbiome differences between benign and malignant conditions is essential. In this study, bile samples were collected aseptically from 141 patients with biliary tract cancer (BTC) or benign biliary diseases (BBDs) who underwent endoscopic retrograde cholangiopancreatography or biliary tract surgery. Quality control PCR was performed to amplify the V3–V4 region of the bacterial 16S rRNA gene. Metagenomic sequencing of bile was successfully performed in 35 of 56 samples collected from patients with BTC and 24 of 85 samples from patients with BBD. The mean alpha diversity values comprised 2.788 ± 2.833 and 2.319 ± 1.355 in the BBD and BTC groups, respectively (*p* = 0.399). The bacterial species (4.7%) were shared between groups, whereas 12.3% and 83% were indicated to patients with BTC and BBD, respectively. *Bacteroides coprocola*, *Prevotella copri*, and *Bacteroides plebeius* were more frequently identified in the bile of patients with BTC, whereas *Bacteroides vulgatus* and *Bacteroides uniformis* were more abundant in the bile of patients with BBD. Distinct patterns of microorganism abundance between the two groups of patients suggest association of bile microbiome with disease status, so its diagnostic potential should be validated in further studies.

## 1. Introduction

The human microbiome is a complex and dynamic ecosystem that plays a vital role in maintaining health and contributing to disease development. Among its components, the gut microbiota is one of the most extensively studied microbial communities, owing to its profound influence on host physiology. It is now well established that the composition of intestinal microorganisms regulates diverse physiological processes, including immune system modulation, metabolic functions, and the gut–brain axis [1,2]. Disruptions in microbial homeostasis, known as dysbiosis, have been implicated in the pathogenesis of various conditions, such as inflammatory bowel disease, metabolic syndrome, obesity, and various types of cancer [3,4].

Bile, a digestive fluid primarily known for its role in fat emulsification, has long been considered sterile, owing to its antimicrobial properties. However, recent advances in sequencing technologies have revealed that bile contains diverse bacterial communities, challenging this assumption [5]. These microorganisms play prominent roles in benign and malignant conditions affecting the biliary tract. For example, emerging evidence suggests that specific microbial signatures in bile are associated with BTC, potentially contributing to tumor development and progression through mechanisms such as inflammation and bile acid metabolism [6,7].

The distinction between the bile microbiome in benign and malignant conditions remains poorly understood but is critical for advancing our understanding of its role in health and disease. Comparative analyses of bile microbial profiles across different pathological states could provide valuable insights into the microbial contributions to disease mechanisms and help identify potential biomarkers for early detection or therapeutic targets. By elucidating the complex interplay between the bile microbiome and host factors, we can better understand the pathogenesis of biliary diseases and develop novel strategies for their prevention and treatment. Therefore, the present study aimed to characterize and compare the bile microbiomes of patients with BTCs and those with BBDs.

## 2. Materials and Methods

### 2.1. Patient Sample Collection

We enrolled 143 patients at the Pusan National University Hospital between May 2021 and December 2023, including 56 patients diagnosed with BTC and 87 patients with BBD. Eighty-four patients were excluded because of their unsuitability for sequencing analysis. Clinical data and bile samples were collected during surgery and endoscopic retrograde cholangiopancreatography (ERCP).

All bile samples obtained during ERCP were collected using a new sterile catheter immediately after guide wire-assisted selective biliary cannulation and prior to endoscopic sphincterotomy only in cases where the ampullary orifice was patent. To minimize contamination, bile was aspirated directly from the bile duct using a sterile syringe suction before the administration of contrast agents or any therapeutic intervention.

A minimum of 5 mL of bile was collected, and efforts were made to obtain 10 mL or more whenever feasible. Fresh bile samples were immediately transferred into sterile containers and stored at −80 °C after collection. Subsequently, 1 mL aliquots were divided into separate sterile tubes for downstream analysis. These procedures were performed to minimize environmental exposure and external bacterial contamination during sample collection and processing. All experiments were performed in accordance with the Declaration of Helsinki and relevant ethical guidelines and regulations. The study was approved by the Institutional Review Board of the Pusan National University Hospital (approval No. 2105-004-102). Written informed consent was obtained from all participants prior to their enrollment in the study.

### 2.2. Bile Bacterial DNA Extraction and 16S rRNA Gene PCR

Frozen 1 mL bile juice samples were thawed, and microbiome DNA was extracted using the QIAamp DNA Microbiome Kit (Qiagen, Hilden, Germany) according to the manufacturer’s instructions. The extracted microbiome DNA was eluted in 50 µL of the kit’s elution buffer and stored at −20 °C until further analysis.

Initial quality control (QC)-PCR for bacterial 16S rRNA sequencing was performed to amplify the V3–V4 regions of the bacterial 16S rRNA gene (Supplementary Table S1). The PCR products were assessed for the V3–V4 region by electrophoresis. Samples with confirmed V3–V4 region expression were subjected to a second QC step, followed by 16S rRNA gene sequencing using 3BIGS (Hwaseong-si, South Korea).

A second QC-PCR was performed using the same primers and conditions as those used for the first QC-PCR. PCR bands from the second QC-PCR were identified by electrophoresis. PCR products were purified by magnetic isolation using AMPure beads. The concentrations of the purified products were measured using a Nanodrop spectrophotometer, followed by Index PCR (Supplementary Table S2). The Index PCR products were isolated using a magnetic method with the AMPure Kit. Subsequently, DNA pooling was performed, and the pooled DNA was analyzed by gel electrophoresis.

### 2.3. 16s rRNA Sequencing and Analysis

The V3–V4 hypervariable regions of the 16S rRNA gene were targeted for PCR amplification using barcoded universal primers. This region was selected for sequencing to maximize taxonomic resolution and ensure reliable community profiling. This region provides superior discriminatory power and higher alpha diversity estimates compared to those afforded by other hypervariable regions, making it the most suitable target for accurate species-level identification [8,9,10]. Sequencing was performed on the Illumina MiSeq platform (Illumina Inc., San Diego, CA, USA) in strict accordance with the manufacturer’s protocols. Raw sequencing data were processed using the QIIME2 bioinformatics framework (v.2022.02). To enable downstream analysis, 300 bp reads were demultiplexed and merged to reconstruct full-length sequences for each specimen. Sequence QC was performed using the DADA2 plugin to generate high-resolution amplicon sequence variants (ASVs) with forward and reverse reads truncated to 282 and 239 bp, respectively. This pipeline generated 5,927,608 sequences across 59 samples, identifying 6547 unique features with an average sequencing depth of 100,467 reads per sample. A comprehensive feature table was established to profile taxonomic composition, assess alpha and beta diversity metrics, and detect differentially abundant features across experimental groups. Taxonomic assignment of ASVs was achieved using a sklearn-based classifier trained on the SILVA database (version 138).

Alpha diversity of the bile microbiome was assessed using the Shannon diversity index, which reflects both taxonomic richness (number of observed taxa) and evenness (relative abundance distribution of taxa). The Shannon index was calculated from ASV relative abundances generated using the DADA2 pipeline implemented in QIIME2.

The Shannon diversity index is one of the most widely used and validated metrics for evaluating the within-sample microbial diversity in 16S rRNA gene–based microbiome studies. It is particularly suitable for comparing the microbial community complexity between clinical groups and has been extensively applied in studies of gastrointestinal and biliary microbiomes. Accordingly, it was selected as the primary measure of alpha diversity in this study.

## 3. Results

### 3.1. Patient Characteristics

**Figure 1:**
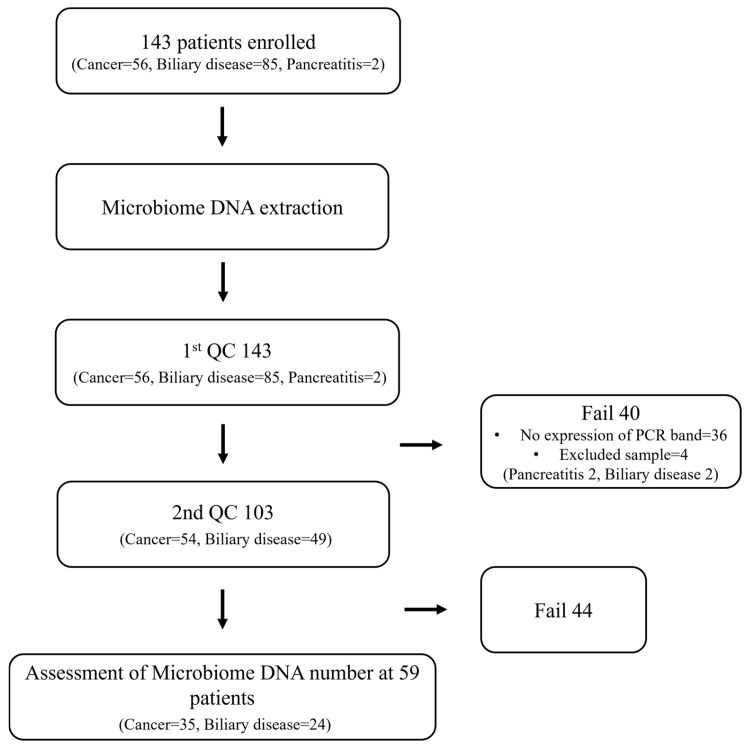
Flowchart of study. A total of 143 patients were enrolled, of whom 59 were finalized for the QC analysis.

In total, 141 bile samples were collected, 56 from patients with BTC and 85 from patients with BBD, following the exclusion of two patients with pancreatitis. (Supplementary Table S3). After the first QC step, 103 samples (73.05%) passed, with 54 BTCs (96.4%) and 49 BBDs (57.6%) samples meeting the quality criteria. Following the second QC step, 59 samples (41.84%) were successfully analyzed, including 35 BTCs samples (62.5%) and 24 BBDs samples (28.2%). An overall design of the study is shown in a flowchart (Figure 1). Table 1 presents the baseline patient characteristics. A total of 59 patients (35 BTC, 24 BBD) were included in the study. The median age of the patients with BTC was 72 years (range: 54–89), which was significantly higher than that of the patients with BBD (64 years, range: 39–89, *p* = 0.002). The proportion of male patients was similar between the two groups (34.3% vs. 50%, *p* = 0.311). There were no significant differences between the groups in terms of hypertension (*p* = 0.776), diabetes (*p* = 0.337), hepatitis B infection (*p* = 0.187), or menopause rate (*p* = 0.263). Smoking status, liver fluke infection, and freshwater fish intake were not significantly different between groups. Among the 35 patients with BTC, the most common diagnosis was extrahepatic cholangiocarcinoma (EHCC) (n = 17), followed by ampulla of Vater carcinoma (AoV ca) (n = 7), perihilar cholangiocarcinoma (perihilar CCC) (n = 7), intrahepatic cholangiocarcinoma (IHCC) (n = 1), and gallbladder carcinoma (GB ca) (n = 3). Among the 24 patients with BBD, the most frequent diagnosis was chronic cholecystitis (n = 13), followed by common bile duct (CBD) stones (n = 7), adenomyomatosis (n = 2), and gallbladder (GB) polyps (n = 2).

### 3.2. Taxonomy and Diversity

**Figure 2:**
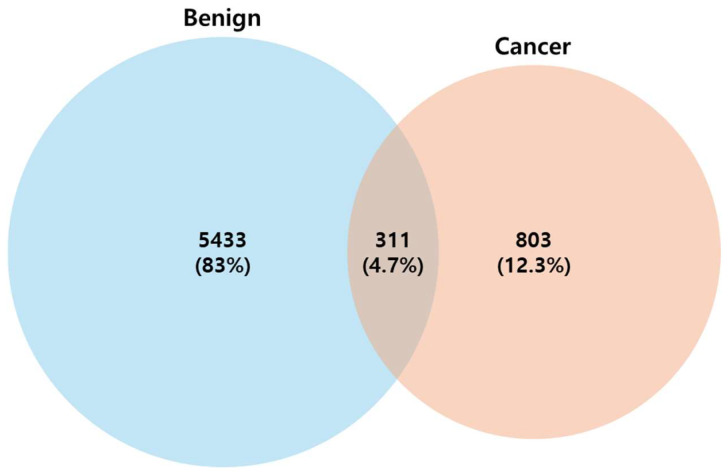
Taxonomy Venn diagram showing core microbiome numbers and percentages in BTCs (cancer) and BBDs (benign).

**Figure 3:**
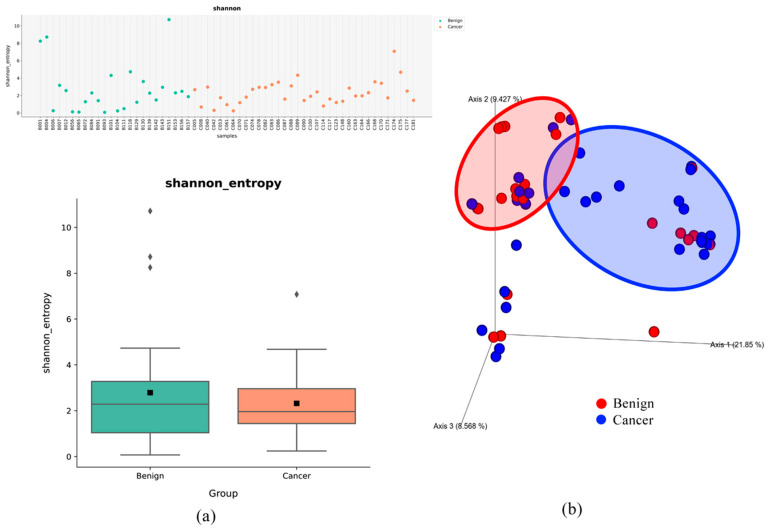
Alpha diversity and beta diversity. (**a**) In BTCs and BBDs, microbial alpha diversity was identified using the Shannon diversity index. BBDs group (benign, 2.788 ± 2.833), BTCs group (Cancer, 2.319 ± 1.355), *p* = 0.399; (**b**) beta diversity was visualized as a PCoA plot using the Bray–Curtis dissimilarity index. (Red point: Benign, BBDs, blue point: cancerous BTCs).

The microbiome taxonomy in bile overlapped between patients with BTC and those with BBD, with 311 species (4.7% of the total number of microorganisms analyzed) detected in both groups (Figure 2). Although 803 species were unique to the samples from patients with BTC, they accounted for only 12.3% of the total microbial abundance in bile. In contrast, 5433 species were unique to the samples of patients with BBDs, representing approximately 83% of the total microbial community analyzed. This suggests that a relatively small number of dominant species comprises most of the bile microbiome, whereas unique taxa tend to be low in abundance (Figure 2). The alpha and beta diversities are shown in graphs (Figure 3). Although alpha diversity, assessed using the Shannon method, did not show significant differences, there was a tendency for an increase in bile content in patients with biliary tract diseases. In contrast, the beta diversity was not significantly different between the groups. The alpha diversity values were as follows: BBDs group (2.788 ± 2.833) and BTCs group (2.319 ± 1.355), with a *p*-value of 0.399.

### 3.3. Linear Discriminant Analysis Effect Size (LEfSe) and Phylogenetic Tree Analysis

**Figure 4:**
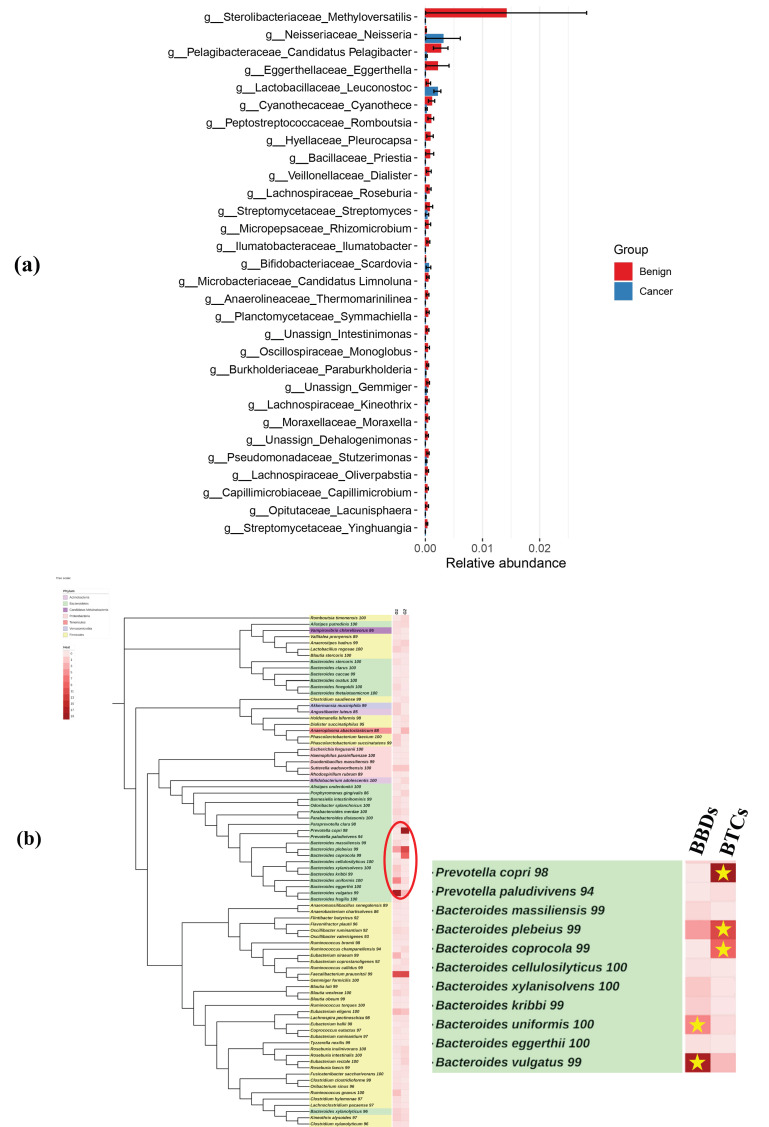
Analysis of the relative abundance of microbial taxa between BBDs and BTCs groups. (**a**) LEfSe analysis at the genus level, used to identify the distinguishing characteristics between the two groups (red bar: Benign, BBDs, blue bar: Cancer, BTCs). (**b**) Phylogenetic tree of microbial taxa at the phylum level. Bacteria taxa that were significantly increased in more than 13 samples in either group are indicated with a yellow star. LEfSe: Linear discriminant analysis Effect Size.

Figure 4 presents the results of Linear discriminant analysis Effect Size (LEfSe) analysis at the genus level, highlighting significant differences in the relative abundance of microbial taxa between the BBDs and BTCs groups. Among the identified genera, *Sterolibacteriaceae Methyloversatilis* exhibited the most significant differences. Additionally, phylogenetic tree analysis revealed that *Bacteroides coprocola*, *Prevotella copri*, and *Bacteroides plebeius* were more frequently detected in the bile from patients with BTCs, whereas *Bacteroides vulgatus* and *Bacteroides uniformis* were more commonly observed in the bile of patients with BBDs.

## 4. Discussion

The present study identified notable differences in the bile microbiome of patients with and without BBDs. Although alpha diversity was lower in patients with BTCs than in those with BBDs, this difference was not statistically significant (*p* = 0.399). Taxonomic analysis revealed that 4.7% of the bacterial species were shared between the two groups, with approximately 12.3% unique to patients with BTCs and 83% specific to BBDs. Specific bacterial taxa were differentially abundant, with *Bacteroides coprocola*, *Prevotella copri*, and *Bacteroides plebeius* being more prevalent in the bile of patients with BTC, whereas *Bacteroides vulgatus* and *Bacteroides uniformis* were more frequently detected in BBDs, as shown in the phylogenetic tree analyses. Furthermore, *Sterolibacteriaceae Methyloversatilis* was more prevalent in the bile of BBDs patients, according to LEfSe analysis.

Alpha diversity refers to the within-sample diversity of microbial communities and measures the species richness and evenness in a single sample. It provides insights into the complexity of a microbial ecosystem by assessing indices, such as the Shannon and Simpson diversity indices [11]. In contrast, beta diversity, assesses between-sample diversity, comparing microbial composition across different reference groups to identify variations in community structure [12]. A decrease in alpha diversity indicates reduced richness and evenness of the microbial species within a sample, suggesting diminished microbial complexity and potential dysbiosis [13]. Such reductions have been associated with various disease states, including cancer, as lower microbial diversity may lead to impaired microbial ecosystem stability and altered host–microbe interactions. Previous studies have also reported decreased microbial diversity in patients with cholangiocarcinoma, confirming a reduction in alpha diversity [14]. Similarly, although not statistically significant, our study identified a decreasing trend in alpha diversity.

Our study identified significant differences in bile microbiomes between patients with and without BBDs. To explore whether these differentially abundant microorganisms had a causal relationship with BTC, we conducted a literature review on the functional roles of these taxa.

*Prevotella copri*, which is enriched in the bile of patients with BTCs, is frequently associated with liver cirrhosis and systemic inflammation [15,16]. Previous studies have suggested that *P. copri* promotes inflammation by generating lipopolysaccharides (LPS), which activate Toll-like receptor 4 (TLR4) signaling [16,17]. The chronic activation of inflammatory pathways may serve as a risk factor for the long-term development of cholangiocarcinoma. Additionally, *P. copri* has been associated with increased susceptibility to rheumatoid arthritis and insulin resistance, indicating its role in systemic inflammation [17].

Similarly, *Bacteroides plebeius* has been implicated in the impairment of the intestinal barrier function, which may indirectly disrupt bile acid homeostasis [18,19]. Given that cholangiocytes rely on bile acids for cellular protection, this bacterium may contribute to malignancy by disrupting bile acid homeostasis and potentially reducing the protective function of cholangiocytes.

*Bacteroides coprocola*, another taxon enriched in the bile from patients with BTC, has been implicated in metabolic dysfunction when depleted, leading to fatty liver disease and influencing hepatocytes and cholangiocytes [20,21]. Although the effects of this reduction have been documented previously, in the present study, this bacterium was upregulated in patients with BTC. Therefore, the effect of this increase remains unclear.

Conversely, some bacterial taxa that were enriched in patients with BBD were reduced in patients with BTC. *Bacteroides uniformis*, for instance, is involved in bile acid metabolism, helping to convert hydrophobic bile acids into hydrophilic forms, thereby reducing bile acid toxicity [22]. The depletion of *B. uniformis* in patients with BTC may lead to the accumulation of hydrophobic bile acids, which could negatively affect cholangiocytes and contribute to carcinogenesis. *B. uniformis* has been shown to alleviate liver inflammation by decreasing pro-inflammatory cytokines such as TNF-α, IL-22, IFN-γ, and IL-10, leading to the attenuation of hepatic inflammatory responses [23]. Additionally, it lowered LPS levels, thereby suppressing TLR4 signaling and mitigating inflammation-induced liver damage, suggesting its potential as a therapeutic candidate for inflammatory liver diseases.

Additionally, *Bacteroides vulgatus*, which was more abundant in patients with BBDs, is known to be a 7α-dihydroxylation bacterium that plays a role in secondary bile acid metabolism [24]. This species contributes to gallstone formation by producing toxic bile acids. Given that many patients with BBD in our cohort had gallstones or gallbladder-related conditions, the increased presence of *B. vulgatus* in this group was probably reflective of their disease state rather than a direct link to malignancy. Notably, *B. vulgatus* is associated with insulin resistance and type 2 diabetes, suggesting its involvement in metabolic disorders.

Via LEfSe, *Sterolibacteriaceae Methyloversatilis* was identified as a significant taxon in the BBDs group. However, this bacterium is primarily recognized as an environmental microorganism, rather than a common constituent of the human microbiome. The detection of *S. Methyloversatilis* in bile samples raises important questions regarding its origin and potential roles. However, it remains unclear whether its presence reflects a true biological function or is the result of external contamination during sample collection and processing. Further research is needed to determine whether this bacterium plays a functional role in the biliary environment or whether its presence is merely an artifact of contamination. Future studies incorporating metagenomic and functional analyses are crucial to elucidate their potential involvement in biliary physiology and disease processes.

Although our findings suggest potential microbial influences on biliary tract carcinogenesis, a direct causal relationship between these taxa and BTCs remains unclear. Although some bacteria in patients with BTC have been better understood in previous studies, others remain unclear, and the same applies to patients with BBDs.

In the present study, a substantial proportion of bile samples, particularly those from patients with BBDs, failed to yield sufficient bacterial DNA for 16S rRNA sequencing. This discrepancy could be attributed to several factors. First, the microbial biomass in bile is inherently low, and benign conditions, such as gallstones or cholecystitis, may involve fewer or more transient microbial colonizers than malignant diseases. Second, although bile samples were collected under aseptic conditions, subtle contamination during ERCP or surgical handling, delays in processing, and insufficient sample volume may have contributed to microbial degradation or PCR inhibition. Moreover, standard PCR-based quality control may not be sufficiently sensitive to detect low-abundance bacterial DNA, resulting in the exclusion of borderline-quality samples.

To improve microbial detection and minimize contamination, future studies should implement improved sterile techniques, such as the use of double-sheathed collection catheters, immediate in-procedure cryopreservation, and closed-system handling, to reduce environmental exposure. Additionally, optimizing DNA extraction protocols for low-biomass fluids and incorporating shotgun metagenomics or whole-genome amplification may improve the microbial yield and diversity resolution. Finally, the routine inclusion of negative controls and contamination-aware bioinformatics pipelines is essential for distinguishing true bile-resident microbes from environmental noise, especially in studies involving low-microbial biomass samples, such as bile.

Our study had several limitations. First, it was not designed to establish a definitive causal role of individual bacterial taxa in biliary tract tumorigenesis. Given the descriptive nature of taxonomy-based 16S rRNA sequencing and limited sample size, this study should be interpreted as a hypothesis-generating investigation to identify microbiome patterns associated with malignant versus benign biliary conditions. Second, this study was conducted at a single institution with a relatively small sample size, so its findings may have limited generalizability. Larger multicenter studies are necessary to validate these results in diverse populations. In addition, although the absence of a healthy control group is a limitation, the use of the benign control group is a widely accepted standard in biliary research because of the ethical concerns and procedural risks associated with invasive bile collection from healthy individuals [25]. Third, although bile was collected aseptically, the possibility of contamination during ERCP or other surgical procedures cannot be entirely excluded. Although the alpha diversity analysis indicated that ERCP did not significantly affect the microbiome, further studies with strict contamination controls are required to confirm these findings. Fourth, this study primarily focused on the taxonomic differences in bile microbiome using 16S rRNA sequencing. However, functional insights into the metabolic pathways and mechanisms by which these bacteria influence disease progression remain elusive. Future studies incorporating metagenomics, transcriptomics, and metabolomics will provide deeper insights into the functional roles of these microorganisms. Fifth, the cross-sectional study design limited the ability to establish causality between bile microbiota alterations and BTC. Longitudinal studies are required to determine whether changes in the microbiome precede cancer development or are a result of disease progression. Sixth, potential confounding factors, such as antibiotic exposure, diet, and underlying comorbidities, may influence bile microbiota composition. Although strict dietary control was not enforced, all bile samples were collected after fasting for at least 8 h to minimize acute dietary influences. Moreover, our findings reflected the biliary microbiome in the real-world clinical conditions, which may enhance the generalizability of our results for future clinical applications and diagnostic modeling. However, the potential impact of long-term dietary habits remains a limitation that warrants further investigation in future studies. Bile acids play crucial roles in shaping the microbiome and may influence cancer development. However, the present study did not include a comprehensive bile acid analysis. Future studies should integrate bile acid profiling to explore the interplay between microbial composition and disease pathogenesis. Lastly, although no significant difference in the overall microbial diversity was observed between the BTCs and benign biliary disease groups, the benign controls may already represent a high-risk population, limiting their ability to detect cancer-specific changes. However, species-level analysis identified certain taxa, such as *Bacteroides coprocola* and *Prevotella copri*, as being more abundant in patients with BTC, suggesting a potential role in carcinogenesis. However, we did not include experimental validation of this relationship in this study. As BTC is an orphan disease, acquiring an adequate sample volume from a large cohort within a standard research timeframe is extremely challenging. The data presented here establish key microbial profiles that warrant further investigation. In future studies, we aim to verify the relationships between altered abundance of various microorganisms and the development of BTC and other pathological conditions involving bile ducts and explore their clinical applicability through a multicenter collection study over the next 5 years.

## 5. Conclusions

Our study revealed a trend toward decreased microbial diversity in the bile of patients with BTC. *Prevotella copri*, *Bacteroides plebeius*, and *Bacteroides coprocola* were more abundant in patients with BTC, whereas *Bacteroides uniformis* and *Bacteroides vulgatus* were more prevalent in patients with BD. However, the precise effects of these bacteria on disease progression remain unclear. Further studies, including longitudinal investigations with larger cohorts and bile acid metabolic analyses, are necessary to elucidate the precise roles of these microbiota in BTC.

## Figures and Tables

**Table 1:** Baseline characteristics of patients.

Characteristics	Total (n = 59)	BTCs (n = 35)	BBDs (n = 24)	*p*-Value
Age, yr	69 (42–89)	72 (54–89)	64 (39–89)	0.002
Sex, Male (%)	24 (40.7)	12 (34.3)	12 (50)	0.311
Hypertension	26 (44)	16 (45.7)	10 (41.7)	0.776
Diabetes	14 (23.7)	10 (28.6)	4 (16.7)	0.337
Hepatitis HBV/HCV	2 (3.4)/0 (0)	0 (0)/0 (0)	2 (8.3)/0 (0)	0.187
Menopause	18 (51.4)	10 (43.5)	8 (66.7)	0.263
Smoking none/current/ex-	34 (57.6)/18 (30.5)/7 (11.9)	19 (54.3)/11 (31.4)/5 (14.3)	15 (62.5)/7 (29.2)/2 (8.3)	0.612
Infection with the liver fluke	4 (6.8)	3 (8.6)	1 (4.2)	0.602
Intake of Freshwater fish	14 (23.7)	9 (25.7)	5 (20.8)	0.658
Disease entity		EHCC 17 AoV ca 7 Perihilar CCC 7 IHCC 1 GB ca. 3	CBD stone 7 Chronic cholecystitis 13 adenomyomatosis 2 GB polyp 2	N/A

EHCC: extrahepatic cholangiocarcinoma; AoV ca: ampulla of Vater carcinoma; Perihilar CCC: perihilar cholangiocarcinoma; IHCC: intrahepatic cholangiocarcinoma; GB ca: gallbladder carcinoma; GB polyp: gallbladder polyp; N/A: Not applicable.

## Data Availability

The data presented in this study are available on request from the corresponding author due to privacy and ethical restrictions, as the data contain sensitive information that could compromise the privacy of research participants.

## Supplementary Materials

The following supporting information can be downloaded at: https://www.mdpi.com/article/10.3390/microorganisms14010208/s1, Table S1: PCR primers and conditions for amplification of the V3-V4 region of the bacterial 16S rRNA gene; Table S2: PCR conditions for 16S rRNA sequencing; Table S3: title Final enrollment and analysis; Figure S1: Result of 1st quality control; Figure S2: Result of 2nd quality control.

## Abbreviations

The following abbreviations are used in this manuscript:

AoV ca	Ampulla of Vater carcinoma
BBD	Benign biliary disease
BTC	Biliary tract cancer
CBD	Common bile duct
EHCC	Extrahepatic cholangiocarcinoma
ERCP	Endoscopic retrograde cholangiopancreatography
GB ca	Gall bladder carcinoma
IHCC	Intrahepatic cholangiocarcinoma
LEfSe	Linear discriminant analysis Effect Size
LPS	Lipopolysaccharides
Perihilar CCC	Perihilar cholangiocarcinoma
QC	Quality control
TLR4	Toll-like receptor 4
