# Supplementary material for: Differences in the Biliary Microbiome Between Biliary Tract Cancer and Benign Biliary Disease
Source: Microorganisms. 2026 Jan 16;14(1):208. doi: 10.3390/microorganisms14010208 (PMC12844354; doi:10.3390/microorganisms14010208)
Supplement: Supplementary file 1 [file microorganisms-14-00208-s001.zip › microorganisms-4050059-supplementary.pdf]

Supplementary Table S1. PCR primers and conditions for amplification of the V3-V4 region of the bacterial 16S rRNA gene.

Primer	Seqeunce (5'-3')	PCR conditions
341 F	CCTACGGGAGGCAGCAG	▣ Initial denaturation : 1 min at 95 ℃<br>▣ PCR (30 cycles)<br>▶ Denaturation: 30 s at 95 ℃<br>▶ Annealing: 30 s at 56 ℃<br>▶ Extension: 45 s at 72 ℃<br>▣Final extension : 5 min at 72 ℃
805 R	GACTACCAGGGTATCTAATCC	

Supplementary Table S2. PCR conditions for 16S rRNA sequencing.

**Component**	**Volume**	Index PCR conditions		
		**Temp**	**Time**	**Cycle**
Second QC PCR product	2.5 μL	96 ℃	3 min	
Nextera Index primer 1 (N7XX)	2.5 μL	96 ℃	30 s	**8 cycles**
Nextera Index primer 2 (S5XX)	2.5 μL	55 ℃	30 s	
2x KAPA HiFi HotStart ReadyMix	12.5 μL	72 ℃	30 s	
D.W	5 μL	72 ℃	5 min	
Total	25 μL	10 ℃	Hold	

Supplementary Table S3. Final enrollment and analysis.

	Number of samples (A)	1st QC	2nd QC (B)	**Analysis (B) / Total (A) (%)**
BTCs	56	54	35	62.5
BBDs	85	49	24	28.2
	141	103	59	41.84

QC: quality control; BTC: biliary tract cancer; BBD: biliary benign disease.

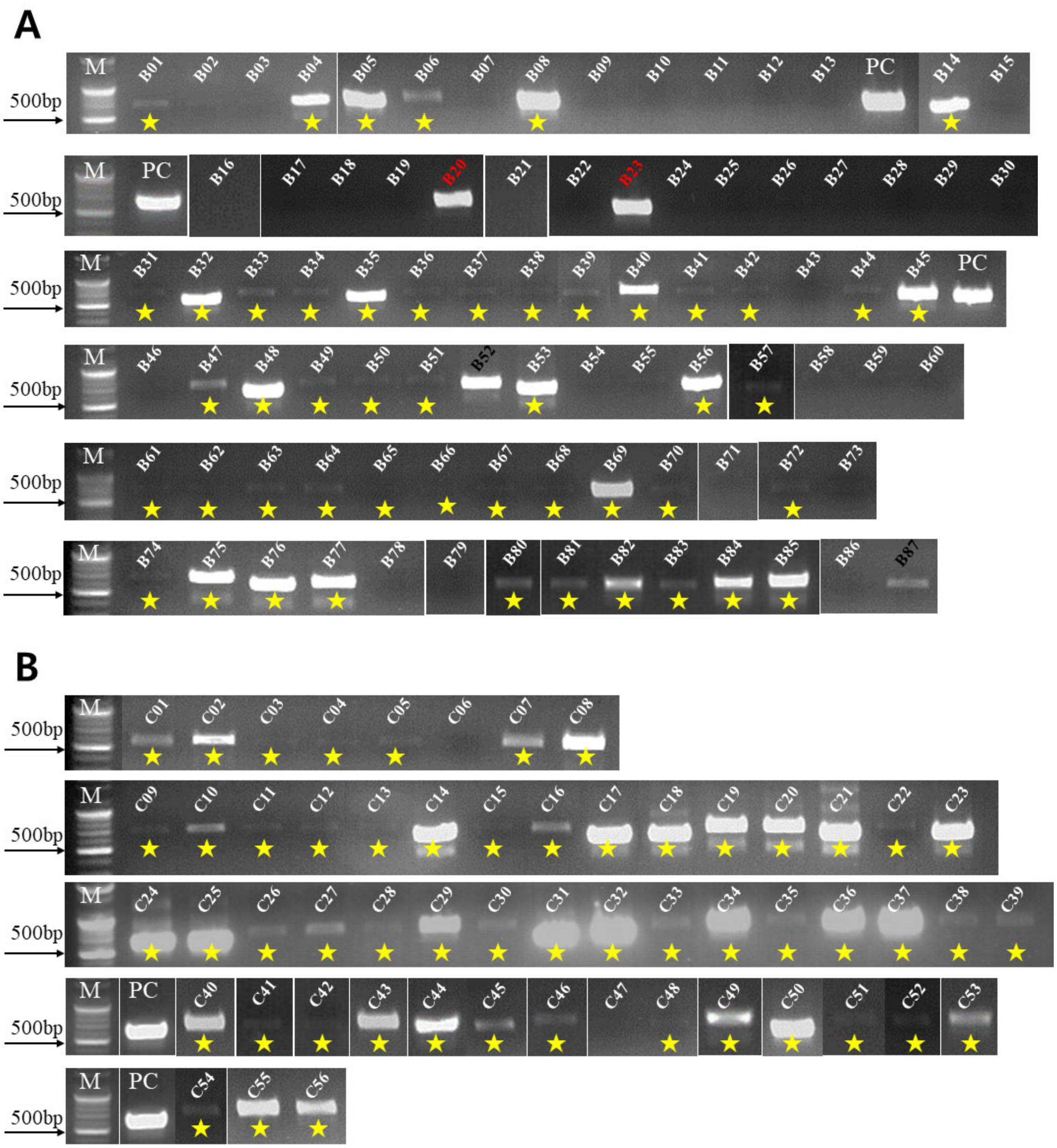

Supplementary Figure S1. Result of 1st quality control .

BBDs (B00 code), B. BTCs (C00 code); the patients in the band expression were all marked as "star". The excluded samples were all marked as “red code; lack of sample’s volume” and “black code; Pancreatitis”.

BTC: biliary tract cancer; BBD: biliary benign disease

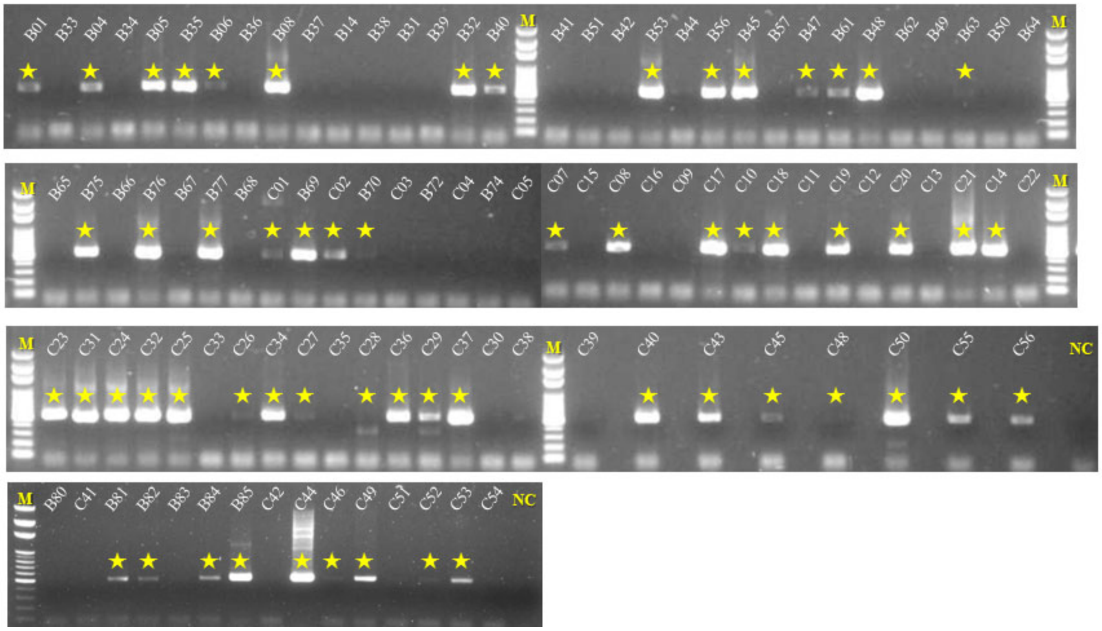

Supplementary Figure S2. Result of 2nd quality control.

BBDs (B00 code), BTCs (C00 code); the patients in the band expression were all marked as "star".

BTC: biliary tract cancer; BBD: biliary benign disease.
